# Inflammatory Mediators Influence the Expression of Nociceptin and Its Receptor in Human Whole Blood Cultures

**DOI:** 10.1371/journal.pone.0074138

**Published:** 2013-09-16

**Authors:** Lan Zhang, Frank Stuber, Ulrike M. Stamer

**Affiliations:** 1 Department of Anaesthesiology and Pain Medicine, Inselspital, University of Bern, Bern, Switzerland; 2 Department of Clinical Research, University of Bern, Bern, Switzerland; University of Würzburg, Germany

## Abstract

**Background:**

Nociceptin/orphanin FQ and its receptor (NOP) are involved in immune responses, inflammation and pain processing. The aim of this study was to investigate the modulation of NOP and prepro-nociceptin (PNoc), the precursor of nociceptin, by inflammatory mediators in human whole blood.

**Methods:**

Peripheral blood from healthy volunteers was cultured for 0, 3, 6 and 24 hrs with or without lipopolysaccharide (LPS), tumor necrosis factor (TNF)-α, interleukin (IL)-1β, IL-10 or interferon (IFN)-γ. NOP and PNoc mRNA of peripheral white blood cells were detected by quantitative RT-PCR. Cytokine concentrations in supernatants of whole blood cultures were measured using ELISA. In addition, an intervention experiment using anti-cytokine antibodies was conducted to evaluate possible mechanisms involved in the modulation of NOP and PNoc by LPS. The primary goal was to investigate NOP and PNoc mRNA expression in human peripheral blood under inflammatory conditions.

**Results:**

LPS significantly suppressed NOP (median area under the mRNA-expression-time curve (1^st^/3^rd^ quartile): 5.4 (4.6/6.6) normalized ratio · hr) and PNoc expression (40.8 (34.4/49.5)) compared to baseline measures (NOP: 22.7 (17.1/25.3); PNoc: 69.9 (58.4/89.2), both p<0.001). LPS incubation induced cytokine concentrations (TNF-α, IL-1β, IL-10 and IFN-γ) in whole blood cultures. Incubation with TNF-α, IL-1β, IL-10 or IFN-γ decreased NOP mRNA levels to varying extents (p<0.05 for all). In contrast, PNoc mRNA expression was decreased by IL-10 only (p = 0.018). The LPS effect on NOP expression could be antagonized by anti-TNF-α and anti-IL-1β, whereas anti-IL-10 and anti-INF-γ had no effect. There was no change of PNoc expression when LPS induced cytokines were antagonized by the respective antibodies.

**Conclusions:**

LPS as well as cytokines suppress mainly NOP and, in part, PNoc mRNA expression in human whole blood cultures. This may represent a negative feedback loop to the previously described upregulation of cytokines by PNoc.

## Introduction

Nociceptin receptor (NOP) is a G-protein-coupled receptor sharing high homology with classic opioid receptors. Nociceptin/orphanin FQ, a 17-amino-acid neuropeptide, is the endogenous ligand of NOP. The nociceptin-NOP system is involved in a wide range of physiological processes, including inflammation and pain [1], [2]. Studies have demonstrated the complex involvement of the nociceptin-NOP activation in pain pathways producing hyperalgesia when administered supraspinally and evoking analgesia when administered at the spinal site [3]. The distribution of NOP and nociceptin and their activity in the immune system suggest that the ligand and its receptor might be regulatory elements in immune modulation [3], [4].

A previous study revealed that nociceptin expression is regulated by injury-induced stimuli [5]. Lipopolysaccharide (LPS) as well as tumor necrosis factor (TNF)-α and interleukin (IL)-1β effect nociceptin expression by activating various signaling pathways in astrocytes [6]. Both NOP and nociceptin were detected in human peripheral blood [7], [8]. Moreover, up- and down-regulation of nociceptin and its receptor in peripheral blood of patients with pain or inflammatory diseases have been reported [9]–[12]. Up to now, numerous studies have extensively investigated the expression of NOP and nociceptin in the nervous system [6], [13]–[16], while much less is known about their modulation in whole blood under inflammatory conditions.

LPS is a major structural feature of gram-negative bacteria and has been found to be a potent inductor of immune responses [17]. Release of some cytokines like TNF-α, IL-1β, IL-10 and IFN-γ is induced in LPS-challenged human whole blood [18]. In the present trial, changes of mRNA expression in NOP and prepro-nociceptin (PNoc), the precursor of nociceptin, in human white blood cells influenced by inflammatory mediators were investigated. The hypothesis was that inflammatory signals modulate NOP and PNoc mRNA expression in human whole blood and that LPS induced effects are mediated by endogenous cytokines.

## Materials and Methods

### Ethics Statement

After approval by the Ethics Committee of the Canton of Bern (KEK 041/09) and participants’ written informed consent, 30 healthy volunteers were enrolled in this observational *ex vivo* study. Exclusion criteria were lack of informed consent, age younger than 18 years, pregnancy, fever, acute infection or a history of inflammatory disease during the last four weeks before the study, as well as any regular intake of medication including antibiotics.

### Blood Culture

All heparinized peripheral blood samples were drawn between 07∶00 and 08∶00 a.m. Undiluted whole blood was cultured in flat-bottomed 24-well culture plates (Greiner Bio-One, Frickenhausen, Germany) at a volume of 900 µl per well prior to the addition of test agents. All agents were diluted with RPMI 1640 medium supplemented with 10% heat-inactivated fetal calf serum, 100 units/ml penicillin and 100 µg/ml streptomycin (all from Sigma, Mannheim, Germany) and prepared freshly from the stock concentration for each experiment. Cultures were incubated at 37°C in a 5% CO_2_ atmosphere. At each indicated incubation time point, cultured cells were harvested, centrifuged at 1500 g for 5 minutes. Subsequently, peripheral white blood cells were collected for RNA isolation, whereas the supernatants were stored at −80°C for further analysis of cytokine concentrations.

The impact of LPS and IL-10 on both NOP and PNoc mRNA expression in peripheral blood cells was initially tested (n = 9). Experiments of dose-dependency effects of LPS and cytokines were performed (n = 4 for each experiment). According to the results of these experiments, blood was cultured using the appropriate concentrations of LPS and cytokines (n = 7). To further investigate the role of the cytokines involved in these cultures, an intervention experiment was conducted (n = 10).

#### Dose - response experiments

Whole blood was analyzed for NOP and PNoc mRNA expression after incubation with or without LPS (6 hrs) and various cytokines (3 hrs). For these dose-response experiments, LPS 0.5–10^4^ pg/ml (from E. coli O127:B8, Sigma, Steinheim, Germany), TNF-α 1–10 ng/ml, IL-1β 1–10 ng/ml, IL-10 0.5–50 ng/ml and IFN-γ 1–20 ng/ml (all from R&D Systems, Oxford, UK) were used.

#### Whole blood cultured with inflammatory mediators

According to the results of the dose-response experiments and findings from previous studies [18], LPS 10 ng/ml, TNF-α 3 ng/ml, IL-1β 3 ng/ml, IL-10 50 ng/ml and IFN-γ 10 ng/ml were used in the subsequent whole blood cultures. Incubation times were set to 0, 3, 6 and 24 hrs. A control group without any additional stimuli was included in each experiment.

#### Intervention experiment

Blood from ten volunteers was used for the intervention experiment. To possibly antagonize the cytokines induced by LPS in whole blood, neutralizing monoclonal antibodies (mAbs) to human TNF-α or IL-1β or IL-10 or IFN-γ or the combination TNF-α+IL-1β (all from R&D Systems, Oxford, UK) were added at a final concentration of 5 µg/ml 30 minutes prior to the treatment of LPS (50 pg/ml). In addition, blood incubated with LPS+isotype Ab 5 µg/ml as well as blood without any stimulant was cultured. Isotype Ab was chosen as control group to estimate the non-specific binding of target antibodies due to crystallisable fragment receptor binding, or other protein-protein interactions according to the immunoglobulin type of the antibodies used in the present study. NOP and PNoc mRNA expression were assessed after the blood was incubated for 3, 6 and 24 hrs with LPS and the respective antibodies.

### RNA Isolation, cDNA Synthesis and Relative Quantification

Total RNA of whole white blood cells collected at the different incubation time points was isolated according to the QIAamp RNA Blood Mini Kit instructions (Qiagen, Hilden, Germany). Briefly, red blood cells were lysed with the erythrocyte lysis buffer; subsequently leucocytes were lysed and homogenized, and the lysate was loaded onto a column for total RNA binding. After wash steps, RNA was eluted and the concentration was determined by optical densitometry at 260/280 nm. cDNA was synthesized using the 1^st^ Strand cDNA Synthesis Kit for RT-PCR avian myeloblastosis virus (AMV) (Roche, Mannheim, Germany) according to the manufacture’s protocol.

The relative mRNA levels of NOP and PNoc were determined by quantitative RT-PCR. Expression of NOP, PNoc and the reference gene human hypoxanthine phosphoribosyl-transferase (HPRT) were detected in separate capillaries as duplicates on a LightCycler 2.0 (Roche, Mannheim, Germany). cDNA prepared from the SK-N-DZ cell line was used as a calibrator.

The cDNA was subjected to PCR amplification in 20 µl reaction volume containing 4 µl of LightCycler® FastStart DNA Master^PLUS^ SYBR Green I (Roche, Mannheim, Germany) and 10 pmol of each primer (TIB MOLBIOL, Berlin, Germany) for NOP and PNoc. For HPRT, 16 nM of universal probe #73 (Roche, Mannheim, Germany), 4 µl LightCycler® TaqMan® Master (Roche, Mannheim, Germany) and 10 pmol of each primer (TIB MOLBIOL, Berlin, Germany) were used. The sequence of primers and PCR conditions have been described previously [7], [10], [19]. Primer sequences were as follows: HPRT, 5′-TGACCTTGATTTATTTTGCATACC-3′ and 5′-CGAGCAAGACGTTCAGTCCT-3′; NOP, 5′-TGCCGTTCTGGGAGGTTATCTA-3′ and 5′-TTAGGGTGAAGGTGCTGGTGA-3′; PNoc, 5′-CCTGCACCAGAATGGTAATG-3′ and 5′-GCTGAGCACATGCTGTTTG-3′. The mRNA expression of NOP and PNoc was analyzed by the LightCycler Relative Quantification Software (Roche Diagnostics, Mannheim, Germany). In the present study, standard curves used to control the efficiency of PCR were generated by PCR amplification of NOP, PNoc and HPRT in a series of diluted cDNA in triplicates, respectively. Data of real-time PCR, including calibrator and samples, were analyzed using the LightCycler Relative Quantification Software. NOP and PNoc mRNA levels were expressed as the target/reference ratio of each sample normalized by the target/reference ratio of the calibrator used in the PCR reactions (normalized ratio) [20].

### Enzyme-linked Immunosorbent Assay (ELISA)

Supernatants of whole blood cultures were collected at pre-defined time points. The amounts of TNF-α, IL-1β and IL-10 protein were measured using human TNF-α/TNFSF1A, IL-1β/IL-1F2 and IL-10 Quantikine ELISA kits (all from R&D Systems, Oxford, UK) according to the manufacturer’s instructions. To detect IFN-γ, the human IFN-γ platinum ELISA kit (eBioscience, Vienna, Austria) was applied. Briefly, supernatants of the cultures were appropriately diluted before proceeding with the ELISA measurement according to the detection range of the assay. Samples were measured in duplicates and a standard curve was created for each plate. The absorbance was read at 450 nm by an ELISA-Reader (Bio-Rad Lab., USA). The results were assessed by calculating the values of coefficient of variation to compare mean concentrations. Minimum detection levels were 15.6 pg/ml for TNF-α, 3.9 pg/ml for IL-1β, 7.8 pg/ml for IL-10 and 0.99 pg/ml for IFN-γ.

### Statistical Analysis

mRNA results are presented as the median normalized ratio (1^st^/3^rd^ quartile) or as the area under the mRNA-expression-time curve (AUC normalized ratio • hr; time points: 0, 3, 6 and 24 hrs). The primary endpoint was the comparison of AUCs of NOP and PNoc mRNA under co-incubation with different stimuli compared to baseline expression. The Mann-Whitney U and the Kruskal-Wallis tests were applied and p<0.05 was considered as statistically significant with results being corrected for multiple testing. Cytokine concentrations are presented as mean±SD and compared by ANOVA.

## Results

### Study Subjects

13 female and 17 male healthy volunteers, aged 26–50 years, participated in the present study.

### Modulation of NOP and PNoc Expression by Inflammatory Mediators

**Figure 1:**
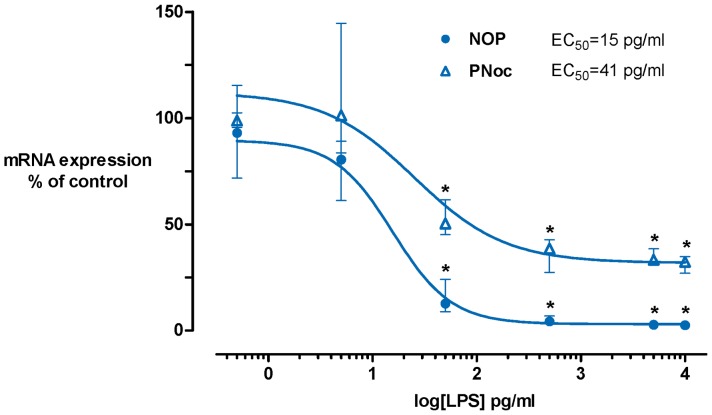
Effects of LPS on NOP and PNoc mRNA expression. Whole blood was cultured without LPS or with increasing concentrations of LPS 0.5–10^4^ pg/ml for 6 hrs. LPS suppressed both NOP and PNoc mRNA expression. Data are presented as medians with interquartile ranges. n = 4, Kruskal-Wallis test with subsequent post hoc analysis, *: p<0.05. LPS, lipopolysaccharide; NOP, nociceptin receptor; PNoc, nociceptin precursor.

NOP and PNoc mRNA were constitutively expressed in peripheral blood cells of all healthy volunteers (median normalized ratio (1^st^/3^rd^ quartile): 1.1 (0.8/1.3) and 5.4 (4.2/6.5)). LPS dose-dependently down-regulated NOP and PNoc expression at a concentration range between 0.5–10^4^ pg/ml with an EC_50_ of 15 pg/ml (logEC_50_ = 1.2±0.1) for NOP and 41 pg/ml (logEC_50_ = 1.6±96.4) for PNoc (Figure 1). The co-incubation of whole blood with pro-inflammatory cytokines (TNF-α, IL-1β and IFN-γ) induced a dose-dependent reduction of NOP mRNA levels.

Results addressing NOP and PNoc mRNA expression under co-incubation with inflammatory mediators calculated as AUC are presented in Table 1. A significant decrease of NOP expression by LPS, TNF-α, IL-1β, IL-10 and IFN-γ was induced compared to the respective controls. LPS effects were most pronounced with a decrease of NOP expression to 12.0 (8.3/25.8)% at 3 hrs and to 5.5 (3.0/13.0)% at 6 hrs compared to controls without co-incubation (both p<0.001). Measures at 3 hrs for IL-10 treated samples were 46.5 (27.8/74.5)%, for TNF-α 31.0 (26.0/41.0)%, for IL-1β 82.0 (59.0/86.0)% and for IFN-γ treated samples 49.5 (43.5/53.5)% compared to controls without co-incubation (all p<0.03).

**Table 1:** AUC of NOP and PNoc mRNA expression (median normalized ratio hr with 1st/3rd quartiles) after whole blood was co-cultured with different stimuli for 0, 3, 6 and 24 hrs.

Stimulation	n	NOP expression	p^a^	PNoc expression	p^a^
**Control without co-incubation**	20	22.7 (17.1/25.3)		69.9 (58.4/89.2)	
**LPS** 10 ng/ml	20	5.4 (4.6/6.6)	0.001	40.8 (34.4/49.5)	0.001
**IL-10** 50 ng/ml	16	12.5 (6.1/16.0)	0.001	51.5 (40.3/59.0)	0.018
**TNF-α** 3 ng/ml	7	10.4 (9.5/12.4)	0.002	48.4 (28.6/65.0)	0.109
**IL-1β** 3 ng/ml	7	14.3 (13.6/17.4)	0.032	50.5 (36.1/73.8)	0.433
**IFN-γ** 10 ng/ml	6	11.1 (10.6/12.1)	0.002	65.8 (53.7/91.1)	0.828

a Comparison to blood cultures without any co-incubation; Mann-Whitney U test, p-values were corrected for multiple testing.

For PNoc expression, a suppression of mRNA expression was only observed for LPS (36.5 (19.3/52.5)% of controls at 3 hrs; p<0.001) and IL-10 (48.5 (42.5/59.0)% of controls; p<0.001). For blood co-incubated with TNF-α, IL-1β or IFN-γ, the level of significance was not met in the AUC analysis (Table 1).

### Concentrations of Cytokines in LPS-induced Whole Blood Cells

To verify LPS effects within the whole blood cultures, cytokine concentrations were measured in culture supernatants. Before stimulation as well as after incubation without stimuli, cytokine concentrations were below the detection limit. After co-incubation with LPS 10 ng/ml for 3 hrs, the concentrations of TNF-α and IL-1β in the supernatants increased. IFN-γ was detectable in the samples after 3 hrs and increased after 6 hrs, whereas IL-10 was detected after 6 hrs of LPS stimulation (Table 2).

**Table 2:** TNF-α, IL-1β, IL-10 and IFN-γ concentrations (pg/ml) in supernatants of whole blood cultures incubated with LPS 10 ng/ml.

Cytokine	Before stimulation	3 hrs	LPS stimulation for 6 hrs	24 hrs
**TNF-α**	<15.6	7077.7±3404.3^a^	**10768.1±4967.6** a	4321.0±3436.7^a^
**IL-1β**	<3.9	915.5±981.7^a^	6392.1±3211.2^a^	**7535.7±3438.5** a
**IL-10**	<7.8	<7.8	224.0±290.2^b^	**3334.0±1210.8** a
**IFN-γ**	<1.0	12.1±16.4	194.4±134.4^a^	**653.9±438.3** a

Data are presented as mean±SD, n = 19. Maximum levels are printed in bold.

a ANOVA p<0.001,

b p<0.05 compared to the respective cytokine concentration before stimulation.

### Interventions

**Figure 2:**
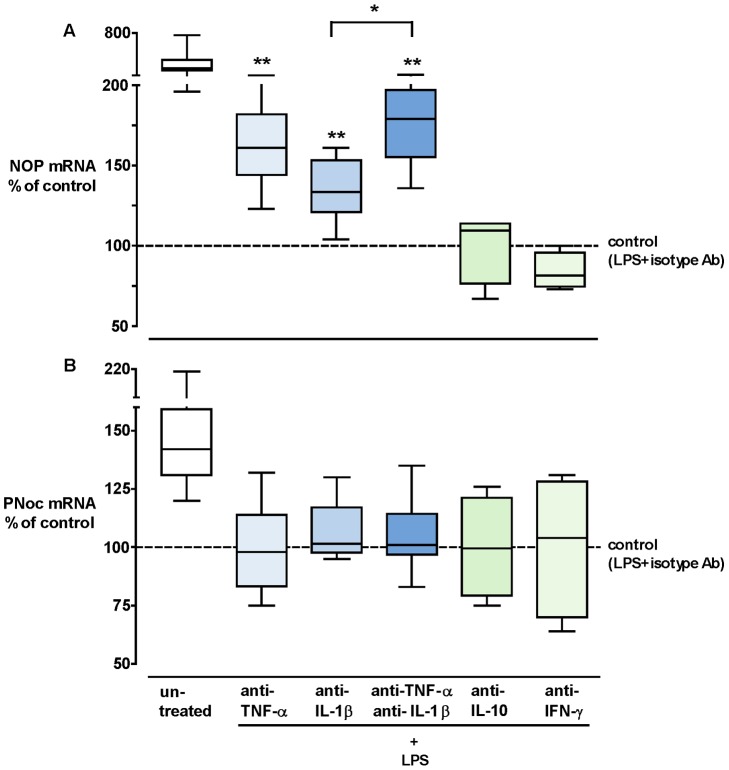
Intervention study. Percent change of NOP (A) and PNoc (B) mRNA expression in whole blood cells after incubation with LPS+anti-TNF-α 5 µg/ml, LPS+anti-IL-1β 5 µg/ml, LPS+anti-TNF-α 5 µg/ml+anti-IL-1β 5 µg/ml, LPS+anti-IL-10 5 µg/ml, LPS+anti-IFN-γ 5 µg/ml, LPS+isotype control 5 µg/ml (dashed line set to 100%) or without any treatment (untreated) for 3 hrs. Box plots show medians (horizontal lines), interquartile ranges (boxes) and 5^th^/95^th^ percentiles (error bars). n = 10, Mann-Whitney U test, *: p<0.01, **: p<0.001, corrected for multiple testing. LPS, lipopolysaccharide; NOP, nociceptin receptor; PNoc, nociceptin precursor; TNF, tumor necrosis factor; IL, interleukin; IFN, interferon.xl.

The effects of anti-TNF-α, anti-IL-1β, anti-IL-10 and anti-IFN-γ mAbs on the expression of NOP and PNoc were investigated in LPS-challenged whole blood (Figure 2). After 3 hrs of incubation, the suppressive effect of LPS on NOP could be partially prevented by co-incubation of antibodies antagonizing TNF-α or IL-1β or both (Figure 2A). Anti-TNF-α, anti-IL-1β and the combination of these two antibodies increased NOP mRNA expression 1.6 (95% CI: 1.2–2.0), 1.4 (1.0–1.6) and 1.8 (1.4–2.1)-fold compared to “LPS+isotype Ab” control. The combination led to a stronger recovery of NOP expression when compared to the group treated with anti-IL-1β only (p = 0.0075), while the difference did not reach statistical significance when compared to the group with anti-TNF-α only.

However, these preventive effects were not observed after 6 and 24 hrs of incubation. As to PNoc expression, none of the anti-cytokine antibodies antagonized the LPS induced down-regulation, neither at 3 hrs (Figure 2B) nor at 6 and 24 hrs.

In summary, the LPS-induced reduction on NOP and PNoc expression was mediated by different cytokines for each target (Table 3).

**Table 3:** Effects of various stimuli on NOP and PNoc mRNA expression in human whole blood.

Stimulation	NOP mRNA	PNoc mRNA
**LPS** 10 ng/ml	**↓↓↓**	**↓↓**
**IL-10** 50 ng/ml	**↓↓**	**↓**
**TNF-α** 3 ng/ml	**↓↓**	→
**IL-1β** 3 ng/ml	**↓↓**	→
**IFN-γ** 10 ng/ml	**↓↓**	→

Suppressive effect: **↓**, ∼25%; **↓↓**, ∼50%; **↓↓↓**, ∼75%; →, no significant change compared to base line.

## Discussion

Previous work underlines the role of the nociceptin-NOP system in inflammatory response and nociceptive pathways [3], [14], [21]–[23]. However, NOP and nociceptin have also been implicated in a wide range of physiological and behavioral functions, e.g. pathways of inflammation, pain, anxiety, fear, stress, tolerance, dependence, locomotion, feeding, learning and memory [1], [3], [10], [12], [24]. Thus, NOP is discussed as promising target of pharmacological research [10], [25]. As NOP and nociceptin are expressed in the human central nervous system as well as in immune cells at similar levels, they are discussed as important mediators of pain perception and immune responses with possible involvement in the brain-immune axis [14], [26]. Understanding the regulation of nociceptin and its receptor NOP is crucial for gaining knowledge and competence to modulate the nociceptin-NOP system. Pain management, modulation of inflammatory processes, e.g. inflammatory disease of the airway [27], may represent future areas in which basic understanding of the activation, expression as well as suppression can be translated to clinical strategies to minimize pain or interfere with inflammation.

### Modulation of NOP and PNoc in Inflammatory Conditions

The current RT-PCR analysis revealed that LPS significantly suppressed NOP and PNoc mRNA expression in human whole blood cells via an LPS-induced upregulation of specific cytokines. Experiments with cytokine antibodies revealed TNF-α and IL-1β as modulators of NOP mRNA expression. Previous findings on NOP and/or nociceptin were inconsistent for other organs: LPS suppressed NOP expression in rats’ basal forebrain and decreased NOP mRNA transcripts in the cerebral cortex, hippocampus and hypothalamus in a severe stressor model [28]. After challenging with staphylococcal enterotoxin A (SEA), NOP and PNoc expression were reduced in spleens of mice, while they were induced in the thymus [24]. On the other hand, nociceptin mRNA was up-regulated by LPS in rat astrocytes and in mice sensory neuron cultures and NOP expression was strongly induced after activation of human lymphocytes by phytohaemagglutinin [13], [15], [26]. Williams and co-workers [7], [8] reported of NOP and PNoc mRNA expression in human peripheral blood mononuclear cells, whereas other authors described NOP mRNA expression on human granulocytes, lymphocytes and monocytes and nociceptin transcripts in resting human peripheral blood lymphocytes, specifically CD19+ B cells [29], [30].

Both *in vitro* and *in vivo* studies have supported the evidence that NOP and nociceptin play a role in systemic inflammatory reactions and sepsis [11]. However, studies in humans have been conflictual: Decreased nociceptin plasma protein has been reported in fibromyalgia-syndrome patients compared to controls [9]. In contrast, protein concentrations were high in acute and chronic pain states as well as in septic patients [12], [31]. A comparison between critically ill patients and healthy controls revealed significant differences in NOP and PNoc mRNA levels in peripheral blood cells [10]. Constitutive NOP mRNA expression was increased in cancer patients, postoperative patients, and septic patients, with highest levels in end-stage cancer patients. In contrast, PNoc expression was reduced in cancer and septic patients but not in postoperative patients [10]. In addition, *ex vivo* findings showed that nociceptin stimulated human monocyte chemotaxis and exerted a modulatory role on T cell functions [4]. All these findings supported the evidence that NOP and nociceptin are regulatory elements in immune response. Taken together, different pathological states and study cohorts or cellular subpopulations and varying measurements (e.g., RNA versus protein) might contribute to the wide range of previous findings. Suppression as well as up-regulation can occur and it is worthwhile to examine underlying mechanisms more closely.

### LPS Model

The LPS model was chosen as LPS induces a variety of pro- as well as anti-inflammatory mediators in human peripheral blood, with TNF peaking at 60 to 90 minutes and IL-1β at 3–4 hrs after initiation of LPS stimulation *ex vivo* as well as *in vivo* [18], [32], [33]. The present ELISA results are in line with these findings, as well as the kinetics of partial prevention of LPS induced NOP suppression with anti-TNF-α or/and anti-IL-1β mAb at 3 hrs. Thus, the intervention experiments support the hypothesis that cytokines like TNF-α, IL-1β are potent modulators of NOP expression in white blood cells induced by LPS. Previous studies have reported that signaling pathways via ERK, p38 and NF-kB are involved in the upregulation of PNoc expression by LPS in rat astrocytes. It has yet to be assessed which signal transduction cascades are involved in human cells. This knowledge can, in turn, be used to interfere with nociceptin activity in vivo.

Culturing blood with LPS for 6 or 24 hrs induced a variety of cytokines, including pro- as well as anti-inflammatory cytokines (i.e. TNF, IL-1β, IFN-γ, IL-10) and other mediators released from peripheral blood cells. The observed inhibition of NOP expression was possibly due to an additive effect of inflammatory mediators in the blood culture at these specific time points. The partial blockade indicated that these cytokines are only part of the pathway responsible for modulation of NOP expression. IL-10, for example, also suppressed NOP; however, this cytokine is induced rather late by LPS. In addition, the binding of the neutralizing antibody possibly resulted in decreased free antibody concentrations at 6 and 24 hrs. These lower antibody concentrations might be unable to completely antagonize newly synthesized cytokines in the cultures. In contrast, the lacking neutralizing effect on LPS-induced PNoc suppression by anti-TNF-α and anti-IL-1β at 3 hrs indicates a differential regulation of NOP and PNoc.

### NOP, PNoc and Cytokines

Gavioli and colleagues [34] summarized in their review that the activation of nociceptin-NOP signaling might induce production of inflammatory mediators, which leads to altered expression of cytokines. The present trial used a reversed approach with LPS/cytokines mediating NOP and PNoc. Interestingly, increase of TNF-α and IL-1β concentrations, but not IL-10 in plasma after administration of nociceptin has been also reported in a rat model with sepsis [35]. As TNF-α and IL-1β mediate most of the physiological disturbances which are characteristic for sepsis, it is possible that the down-regulation of PNoc and NOP in peripheral blood cells might restrict the production of pro-inflammatory cytokines. The observed suppression of PNoc expression is in line with the lower nociceptin precursor mRNA detected in peripheral blood cells from septic patients previously [10]. This might indicate a regulation loop and/or negative feedback between the NOP-system and cytokines.

The mechanisms of immunoregulation and inflammation by cytokines are in part established. However, modulation of PNoc by cytokines has been investigated in animal models and cell lines only [6], [13], [36]. In peripheral blood, cytokine signaling via a restricted number of Jak-Stat pathways positively and negatively regulates all cellular subsets during inflammation [37]. It has to be shown whether similar signal transduction pathways are involved when cytokines exert their effects on PNoc and NOP expression.

Compared to PNoc, NOP appeared to be more sensitive to suppressive effects induced by pro- as well as anti-inflammatory cytokines, at least during early inflammation. Animal studies reported a clear suppressive effect of LPS on NOP in rat CNS [28], whereas staphylococcal enterotoxin A suppressed NOP in the spleen of mice [24]. During the later phases of sepsis (>24 hrs), a variety of cytokines, chemokines and other inflammatory mediators (either derived from peripheral blood cells or secreted into the circulation by organ tissue), might influence the mRNA expression of NOP in blood cells. The comprehensive effect of these inflammatory substances on NOP might be an explanation for NOP mRNA levels detected in peripheral blood from septic patients [10]. Future studies should include an analysis of signal transduction pathways to further elucidate the mechanistic background of this phenomenon and define how the crosstalk between cytokines, NOP and nociceptin works in human peripheral white blood cells.

### Limitations

Some limitations have to be mentioned. First, although LPS is widely used to mimic inflammatory conditions in whole blood cultures, the cultures may not accurately reflect the *in vivo* pathophysiological changes under inflammatory conditions. Second, modulation of NOP and PNoc expression in LPS-challenged whole blood was analyzed only up to 24 hrs, and therefore, expression patterns of NOP and PNoc during the later inflammatory phases are still unclear at this point. Third, no protein was measured. This will be addressed in a future project.

Nevertheless, the present findings support the concept of strong interactions between inflammation and the NOP-system. If NOP ever becomes a therapeutic target, immunotherapy by cytokines can down-regulate it. On the other hand, PNoc appears to be regulated differently: Although LPS showed some effects on PNoc expression, pro-inflammatory cytokines tested here do not contribute to this effect. The different mechanisms of regulating expression of PNoc and NOP have implications when differential modulation of the nociceptin system is considered.

### Conclusions

The present study demonstrated that mRNA expression of the nociceptin receptor and of prepro-nociceptin is modulated differently by inflammatory cytokines in human peripheral blood. LPS and LPS-induced cytokines strongly suppressed NOP, whereas the effects on prepro-nociceptin were minor.
